# Catalysing the monitoring and evaluation of Nationally Determined Contributions through North–South cooperation

**DOI:** 10.1007/s10668-023-03092-5

**Published:** 2023-03-11

**Authors:** F. H. Abanda, E. L. Chia, K. E. Enongene, K. Fobissie, M. B. Manjia, C. Pettang

**Affiliations:** 1 School of the Built Environment, Oxford Brookes University, Oxford, OX3 0BP UK; 2 Fokabs Inc, 955 Rotary Way, Ottawa, Canada; 3 Department of Civil Engineering, National Advanced School of Engineering of Yaoundé, The University of Yaoundé I, Yaoundé, Cameroon

**Keywords:** Climate change, Global South, M&E, NDC, Mitigation, Adaptation

## Abstract

The recent 6th Assessment Report by Intergovernmental Panel on Climate Change has been damning to the world. An overwhelming amount of evidence that Nationally Determined Contributions (NDCs) can contribute to stabilising or reversing the course of impacts of climate change is now common. Given the likely update of NDC measures compounded by their complexities and limited resources, it is imperative to adopt effective Monitoring & Evaluation (M&E) systems to ensure that NDCs achieve their planned objectives. Effective roll-out and M&E of NDCs require full participation from all actors in various countries. However, despite existing evidence that shows the developing countries are the most affected by climate change, the role of their researchers in climate change research is not known. Therefore, the need to investigate the global North–South disparities and develop an agenda for future research about NDCs is imperative. To address this gap, a systematic review was undertaken using appropriate terms in Web of Science, Dimensions and ScienceDirect to identify relevant literature. The analysis of the identified literature led to two main findings. Firstly, most studies about NDCs are conducted by global North research institutes and researchers with very little involvement of those from the global South. Secondly, there is a global paucity of research about M&E of NDCs measures. As a major recommendation, while countries should equitably contribute to rolling out NDC projects, research should play a key role and should be inclusive as possible representing voices from the global North and South.

## Background

The need to limit climate change impacts has led governments to commit to Nationally Determined Contribution (NDC) projects. NDCs are national climate plans highlighting climate actions, including climate-related targets, policies and measures governments plan to implement in response to climate change and as a contribution to global climate action. NDCs are at the heart of the Paris Agreement (Article 4, paragraph 2) where each country is required to outline and communicate their post-2020 climate actions. As of January 2022, according to the NDC Registry, 190 parties have submitted their first NDCs, while only 14 have submitted their second. Based on national circumstances, countries are required to include projects from different sectors that can easily help in achieving their NDCs. Given that only few countries have submitted their second NDCs and that 2020 has just elapsed, many countries are yet to choose eco-friendly projects that can help them meet their climate change obligations. A survey conducted by United Nations Development Programme (UNDP) found that more than two-thirds of the responding countries have either not yet started with planning for NDCs implementation (34%) or are in initial consultations with stakeholders (33%) (UNDP, 2016). A more recent study confirmed that more than 1/3 of National Climate Plans are not easily measured (Ge & Yuan, 2018). However, many countries have underscored the importance of monitoring and evaluation (M&E) systems with 33 countries representing 62% of African NDCs recommending parties to invest in monitoring systems to keep track of climate actions (ADB, 2019). By ratifying the 2015 Paris Agreement of the United Nations Framework Convention on Climate Change (UNFCCC), parties committed themselves to submitting revised NDCs every five years, i.e. 2020, 2025, 2030, etc. The revised NDCs should have an implementation period of 5 years and must be submitted 5 years in advance of the start date for implementation. The Paris Agreement also calls on parties to increase progressively the level of ambition of their NDCs. Given the Paris Agreement was signed in 2015, the implementation of the first NDCs started in 2021. To succeed, know-how and the enabling framework required to put that know-how into practical use is amongst the factors that can aid efficient implementation and monitoring of NDCs (Bakhtiari et al., 2018). It is therefore imperative to have a system, e.g. M&E, in place that can enhance the human capacities for the effective implementation of NDCs. However, there is a paucity of peer-reviewed literature about the evolution or trend of NDCs, NDC sectors and the applications of M&E in NDCs since 2015, when NDC was first conceived. Furthermore, such an M&E system and/or NDCs should be contextual and take into account the specificities of countries or regions concerned. Without being exhaustive, some of the specific differences that may determine the kinds of systems and NDCs or interventions will be discussed. Firstly, there is an overwhelming amount of evidence that developing countries are bearing the highest brunt of impacts from climate change (Pottier et al., 2021; Steynor & Pasquini, 2019), with women and girls disproportionately affected (Khanum, 2021), while developed nations are the greatest negative contributors to climate change. An analysis of national responsibility for historical CO_2_ emissions from 1850 to 2021 showed that the US released more than 509GtCO2 since 1850 contributing to some 20% of the global total and ranked first globally (Evans, 2021). The six highest emitters representing more than 67% of global CO_2_ emissions during the period 1990–2018 are China, the USA, the European Union, India, Russia and Japan (Ortega-Ruiz et al., 2022). Secondly, historical records show atmospheric CO_2_ levels rising from 277 parts per million (ppm) immediately prior to industrialisation in 1750 to 417 ppm by April of 2020, marking a nearly 51% increase (Batrice & Gordon, 2021). Thirdly, a study by Swingle (2019) revealed that historical responsibility of negative contribution to climate change is underemphasised. This differential causes and impacts of climate change require equity to be taken into account in any intervention to mitigate climate change or adapt to it. This view is now being recommended by researchers where they argued for equity to be considered in climate change research (Klinsky et al., 2017; Owen, 2021) and NDCs (Pauw et al., 2017). In the recent COP27 that took place in Egypt, 6–18 November 2022, various governments were critical of the highest CO_2_ emitters (Ortega-Ruiz et al., 2022), who unfortunately were/are lagging in rolling out NDC projects (Argus, 2022; Felter, 2023). While the political pressure on the equitable share of rolling out NDC projects continues especially on the highest global CO_2_ emitters, research in the field is important for further understanding of the performance of such projects. This is important for developing countries who should not feel left out especially given they are those bearing the greatest brunt of climate change impacts.

However, previous research has indicated a huge global North–South divide on climate change and related sustainability concepts research. As far back as 1999 or even prior, studies found that an overwhelming majority of the researchers involved in studies worldwide and possible solutions of/to climate change impact were from industrialised countries, with very few participations from developing countries (Kandlikar & Sagar, 1999). The situation has not changed with recent studies by Hayward and Roy (2019) and Thomas et al. (2020), confirming a paucity of climate change research by those from the developing countries. A detailed analysis by Carbon Brief, has laid bare this disparity or lack of voices from diverse communities. Carbon Brief is a UK-based website specialised in the science and policy of climate change and has won awards for investigative journalism and climate change data analysis. The organisation analysed the “gender” and “country of affiliation” of the authors of 100 highly cited climate science papers from the past 6 years and revealed staggering geographic and gender biases (Tandon, 2021). The study revealed that of the 100 most cited articles, less than 1% of authors in the sample are based in Africa, while almost three-quarters are affiliated with European or North American institutions (Tandon, 2021). With this skewed climate change research with lack of diverse voices, key perspectives are being ignored (Tandon, 2021). For example, as argued by McGrath (2021), issues of concern to African climate researchers are in danger of being ignored.

Despite the efforts by researchers on how to bridge the North–South climate change research divide (e.g. Blicharska et al., 2017), it cannot be achieved without a detail understating about the domain of interest. Therefore, the following research questions are of interest:

- RQ1 What is the trend of NDC research since its inception in 2015?
- RQ2 How involved are those from developing countries involved in NDC research?
- RQ3 What are the main NDC sectors?
- RQ4 Which is the trend and common M&E applications in NDC?

## Nationally Determined Contributions

For a more sustainable future, the historic 2015 Paris Agreement established a goal to limit average global temperature rise to well below 2 °C and to pursue efforts to limit it to 1.5 °C. To meet this set target, every country is expected to prepare and communicate a NDC every 5 years. NDCs include targets, measures and policies, which serve as the basis for national climate action plans. NDCs are at the heart of the Paris Agreement and embody efforts by each country to reduce national emissions and adapt to the impacts of climate change. Prior to the adoption of the Paris Agreement in 2015, more than 160 countries and the European Union publicly outlined what climate actions they intended to take under the global pact, known as Intended NDCs (INDCs). A country’s INDC is converted to an NDC when it formally joins the Paris Agreement by submitting an instrument of ratification, acceptance, approval or accession unless a country decides otherwise. As of January 2022, 190 Parties had submitted their first NDCs and 14 had submitted their second NDCs to the UNFCCC NDC Registry where they outline and communicate their post-2020 climate actions.

The two main categories of NDC interventions are adaptation and mitigation measures. Adaptation is the process of adjusting to the impacts of a changing climate, seeking to moderate or avoid harm and exploit beneficial opportunities. On the other hand, according to the UN Environment Programme climate change mitigation refers to efforts to reduce or prevent emission of greenhouse gases. For example, using new technologies and renewable energies, making older equipment more energy efficient or changing management practices or consumer behaviour. While it is recognised that adaptation is a priority for many developing countries, they will also need to show progress in reducing greenhouse gas emissions. Doing so can have wide benefits, as mitigation actions can be designed to deliver not only emission reductions, but also wider co-benefits in relation to climate change adaptation, development, employment, energy security and public health, for example, (CDKN, 2016). Quite often, the adaptation and mitigation measures are classified according to the various sectors of the economy. To achieve their intended goals, it is imperative to adopt an appropriate M&E system to help with the implementation of the NDCs, to allow for the monitoring and evaluation of NDC adaptation and mitigation measures.

## Monitoring & Evaluation systems and related concepts

Many donor agencies are now insisting on the M&E of NDC projects during their lifecycle for accountability and value for money before they can commit and fund such projects. This is reiterated in Pringle (2011) and WWF (2021) where they argued that organisations that have undertaken adaptation activities or interventions now face the challenge of evaluating what worked, what did not, how and why (Pringle, 2011; WWF, 2021). This is the same situation with those that have undertaken mitigation activities. In the literature “Measurement and Verification” (M&V), M&E, and measurement, reporting and verification (MRV) have been used interchangeable. However, the three concepts are slightly different.

### M&V—Measurement and Verification

Measurement and Verification (M&V) is the process of planning, measuring, collecting and analysing data for the purpose of verifying and reporting energy savings within an individual facility resulting from the implementation of energy conservation measures (EVO, 2022; Team, 2022). According to EVO (2022), M&V activities consist of some or all of the following:

- Metre installation calibration and maintenance.
- Data gathering and screening.
- Development of a computation method and acceptable estimates.
- Computations with measured data, and
- Reporting, quality assurance and third-party verification of reports.

According to Wehnert et al. (2014), the key questions to be answered in M&V are:

- M: What is the exact amount of savings?
- V: Have savings as specified in contract been achieved?

### Monitoring and evaluation systems

To provide a rationale of the relevance of monitoring and evaluation (M&E) system to this proposed study, it is imperative to first define it as used in the aid development field. The International Federation of Red Cross and Red Crescent Societies defines monitoring as “the routine collection and analysis of information to track progress against set plans and check compliance to established standards. It helps identify trends and patterns, adapt strategies and inform decisions for project/programme management” (IFRC, 2013).

The same organisation defines evaluation as “the identification and reflection upon the effects of what has been done, and judging their worth. Findings from an evaluation activity allow project/programme managers, beneficiaries, partners, donors and other project/programme stakeholders to learn from the experience and improve future interventions” (IFRC, 2013). Based on the literature, the definition of M&E system varies depending on the different authors (Bullen, 2018). However, a commonality amongst the various definitions is that M&E system refers to all the indicators, tools and processes that you will use to measure if a programme has been implemented according to plan (monitoring) and is having the desired result (evaluation) (Bullen, 2018). In other words, M&E systems should include things like: who is responsible for M&E tasks in the organisation, the intervals where data should be collected, how the data is collected, who collects the data, the type of database that is used for storing the data, the standard forms and data collection tools to be used, how the data is analysed, the evaluation questions, the frequency with which an evaluation takes place, the budget allocated for evaluation, etc. (Simister, 2009; Vallejo, 2017). A more formal definition of an M&E system is a “series of policies, practices and processes that enable the systematic and effective collection, analysis and use of monitoring and evaluation information (Simister, 2009)”.

A key component of an M&E system is a logic frame that describes a holistic approach in planning, monitoring and evaluating projects or programmes using structured models or matrix forms. Key parameters of log frames are indicators, benchmark data and target. Performance indicators are measures of inputs, processes, outputs, outcomes and impacts for development projects, programmes or strategies (The World Bank, 2004). Benchmark data is a reference data that can be used in measuring the performance of a project or programme against a chosen indicator. For example, one of the indicators of sustainable development goal 1 is poverty level which signifies the number or percentage of individuals living below or above a certain threshold or benchmark. The benchmark for the poverty level was $1.25 a day some years back (WGBC, 2017) and today it is $1.90 a day (World Bank, 2017). A target is where an organisation, country wants to be or what is expected from a project or programme. The benchmark data enable the assessment whether the target was or will be achieved.

### Measuring, reporting and verification

In the context of NDC implementation, the Climate and Development Knowledge Network (CDKN) defines measurement, reporting and verification (MRV) as the process by which countries track and report on the implementation and impacts of mitigation and adaptation actions, and the finance used to support these actions. These three core elements—mitigation, adaptation and finance—can be elements of one integrated, national MRV system or separate MRV systems.

Singh et al. (2016) and MRV Africa (2021) define measurement, reporting and verification as follows:

- Measure: Direct or estimated calculations following strict guidance and protocols. This can include direct measurement using devices or estimation using simple methods or complex models.
- Report: Documentation intended to inform all interested parties. This includes information on methodologies, assumptions and data.
- Verify: Specific procedures or expert review used to verify the quality of the data. Verification can be internal or external.

**Fig. 1:**
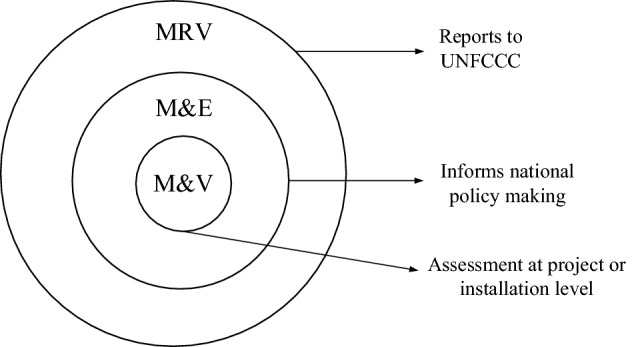
Relationship between the various climate change evaluation measures [Source: Adapted from Wehnert et al. (2014)]

According to Leiter (2017), the difference between verification of adaptation does not have the same relevance internationally than verification of emission reductions. This is because adaptation is more of a country’s specific issue, compounded by a lack of a universal or global unit of measurement. Leiter (2017) further argues that the characteristics of mitigation and adaptation differ significantly and so does the measurement and that MRV should be used for mitigation while M&E for climate change adaptation. Wehnert et al. (2014) summarised the main differences between the three concepts presented in Fig. 1 as follows.

- MRV is used mainly in UNFCCC context—originally used to communicate national GHG emissions (inventories, national communications and biennial update reports)
- M&E is often used for the impact assessment of policies or programmes
- M&V is often used for energy savings of single projects, e.g. in Energy Performance Contracting (e.g. International Performance Measurement and Verification Protocol)

## Research methodology

**Fig. 2:**
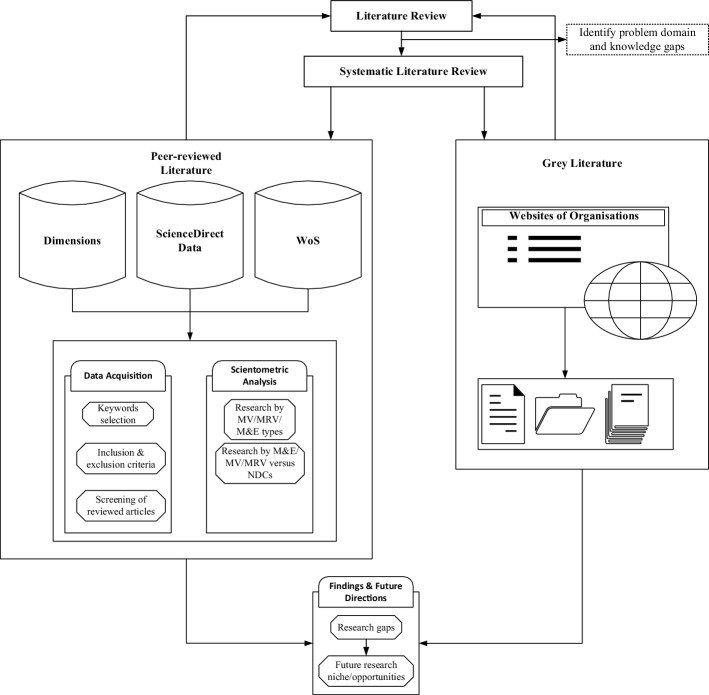
Research method framework

The two main methods used for this study include literature review and systematic literature review. The steps in the two main methods are summarised in Fig. 2.

To facilitate understanding, the details of Fig. 2 will be explained in Sects. 4.1 and 4.2.

### Literature review

The literature review served two main purposes. It was used to gain an in-depth understanding of the NDC projects and related monitoring concepts including M&V, MRV and M&E. Secondly, it was used to identify the gaps in knowledge that underpinned this study. The peer-reviewed literature was obtained from renowned scientific databases such as ScienceDirect and Web of Science. The grey literature was reviewed to develop an understanding of the relevant NDC sectors and mitigation and adaptation measures/projects. The literature from the NDC registry website, National Communications, Technology Needs Assessment reports were examined to identify the projects that can help countries meet their NDCs commitments.

### Systematic literature review

Given this study aims to provide a very meticulous summary of all the available primary research in response to the research questions about NDC projects in relation to climate change, a more systematic and focus approach, also known as systematic review will be used. In research, various kinds of reviews have been used with some selected ones as scoping review (Musonye et al., 2020), mapping review (Al Naimi et al., 2021), umbrella review (Sinkovics et al., 2021) and systematic review (Levenda et al., 2021). Detailed research about 14 typologies of kind of review, which perhaps appear to be a more encompassing study has been covered in Grant and Booth (2009). Although the different review typologies differ with respects to goals, techniques, strengths and weaknesses, there is however, a commonality across most of them. The common factors are inclusion and exclusion criteria, search techniques and search process. Without loss of generality, a systematic review will be used in this study. Based on the method framework for the systematic review presented in Fig. 2, the main category of the documents is peer-reviewed literature. For the grey literature, the documents considered for the review was informed by some selected factors recommended by Ozor et al. (2020) which include:

- International strategic climate change agreements such as the Paris Agreement, UNFCCC, SDGs, AU Charter, Agenda 2063, Malabo Business plan 2012 and UNECA documents, among others.
- Charters and climate change response policies and strategies of Regional Economic Communities (RECs) including Economic Commission for West African Countries, East African Community (EAC), Southern African Development Cooperation (SADC) and Inter-Governmental Authority on Development (IGAD) among others.
- The respective national policies and laws on climate change, NDCs and National Climate Change Action Plans (NCCAPs), policies and other instruments as well as the national policies on green growth among others.
- The national development plans to establish the status of mainstreaming of the NDCs and SDGs in the plans.
- Relevant sectoral policies, laws and regulations in the sectors; and existing tools/mechanisms and institutional arrangements in different countries that facilitate NDCs implementation. The main grey literature was the technology needs assessment (TNA) reports from the TNA database (TNA, 2022). The reports discuss the kind of technologies best suited to a country’s specific climate change situation. The TNA project is funded by the Global Environment Facility and is implemented in close collaboration with the UNFCCC Technology Mechanism.

For the peer-reviewed literature, the Web of Science (WoS), Dimensions and ScienceDirect databases were used. The search period was restricted to the range January 1, 2015–December 31, 2022, and the focus was on journal outputs excluding review articles. The start date of 2015 was chosen because of its significance in climate change studies as it represents the year when NDC was introduced. The queries were conducted on the 29th of May 2022. Depending on the goals of research questions (e.g. RQ1, RQ3, etc.), search terms were formulated and used for the different queries, see Table 1. This approach aligns with guidance from Xiao and Watson (2019), where they proposed that keywords for the search should be derived from the research question(s). The initial outputs were included in the columns with captions 1st output. The outcome is now screened for only research articles with all others including book chapters, conferences articles and peer-reviewed articles excluded. The outcome of this first screening is included in 2nd columns of Table 1. The outputs of the different searches are summarised in Table 1.

**Table 1:** Search terms and output used for analysis

Search term	WoS			Dimensions			ScienceDirect			
	1st output	2nd output	3rd output	1st output	2nd output		1st output	2nd output	3rd output	Main literature on monitoring of NDCs
“Nationally Determined Contributions”	623	561	406	1306	956	503				
“Monitoring and evaluation” and “Nationally Determined Contributions”	1	1		3	2		121	72	60	Visman et al. (2022), Meehan et al. (2019)
“Monitoring and evaluation” and “NDCs”	1	1		3	3		90	57	50	
“M&E” and “NDCs”	0	0		2	2		225	179	119	Gonçalves et al. (2019)
“M&E” and “Nationally Determined Contributions”	0	0		2	1		196	180	130	Leon and Izumi (2022)
“Measurement, reporting and verification” and “Nationally Determined Contributions”	3	3		3	3		34	24	23	0
“Measurement, reporting and verification” and “NDCs”	3	3		3	3		24	15	15	0
“MRV” and “NDCs”	5	5		9	8		71	47	42	0
“MRV” and “Nationally Determined Contributions”	5	5					100	71	65	Usapein and Chavalparit (2017)
“Measurement and verification” and “Nationally Determined Contributions”	0	0		0	0		23	12	12	0
“Measurement and verification” and “NDCs”	0	0		0	0		19	11	11	0
“M&V” and “NDCs”	0	0		0	0		103	76	70	0
“M&V” and “Nationally Determined Contributions”	0	0		0	0		65	55	51	0
Number of articles used for analysis			406			503			648	

01-01-2015 to 31-12-2022, conducted on the 29/05/2022

To determine the global spread of research, WoS and Dimensions were used while Science Direct was used for M&V, M&E and MRV applications on NDCs research. The reasons for this are twofold. The first reason was motivated by the need to minimise the number of duplicates and ensure literature from diverse sources is captured. The three databases WoS, Dimensions and ScienceDirect are produced or hosted by different publishers. WoS is a data source produced by Clarivate Analytics. Dimensions is a data source produced by Digital Science. ScienceDirect is operated by Elsevier. Secondly, the number of outputs from the three databases guided the choice of how they have been used. The term “Nationally Determined Contributions” yielded more results than M&V, M&E and MRV applications on NDCs (4th to the 15th row of Table 1) when used as search terms in Dimensions and WoS. Yet, using M&V, M&E and MRV applications on NDCs (4th to the 15th row of Table 1) in Science Direct yielded more terms than in WoS and Dimensions. As a result, the research for trends of NDC research using “Nationally Determined Contributions” was used in WoS and Dimensions only, while M&V, M&E and MRV applications were used in ScienceDirect. To facilitate analysis, the outputs were analysed using VOSviewer. VOSviewer is a software tool for constructing and visualising bibliometric networks.

## Research findings and discussion

### Direct output

**Fig. 3:**
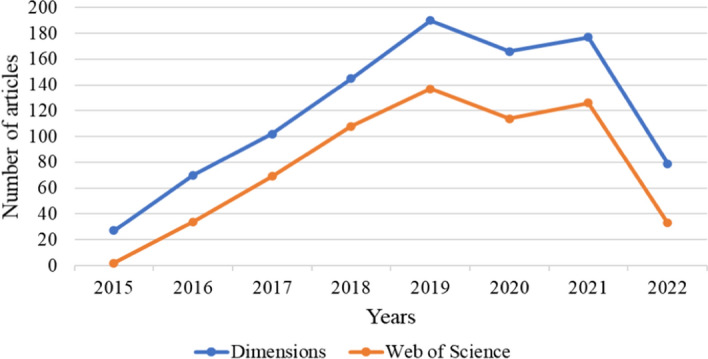
Trend in NDC research outputs (2015–2022)

**Fig. 4:**
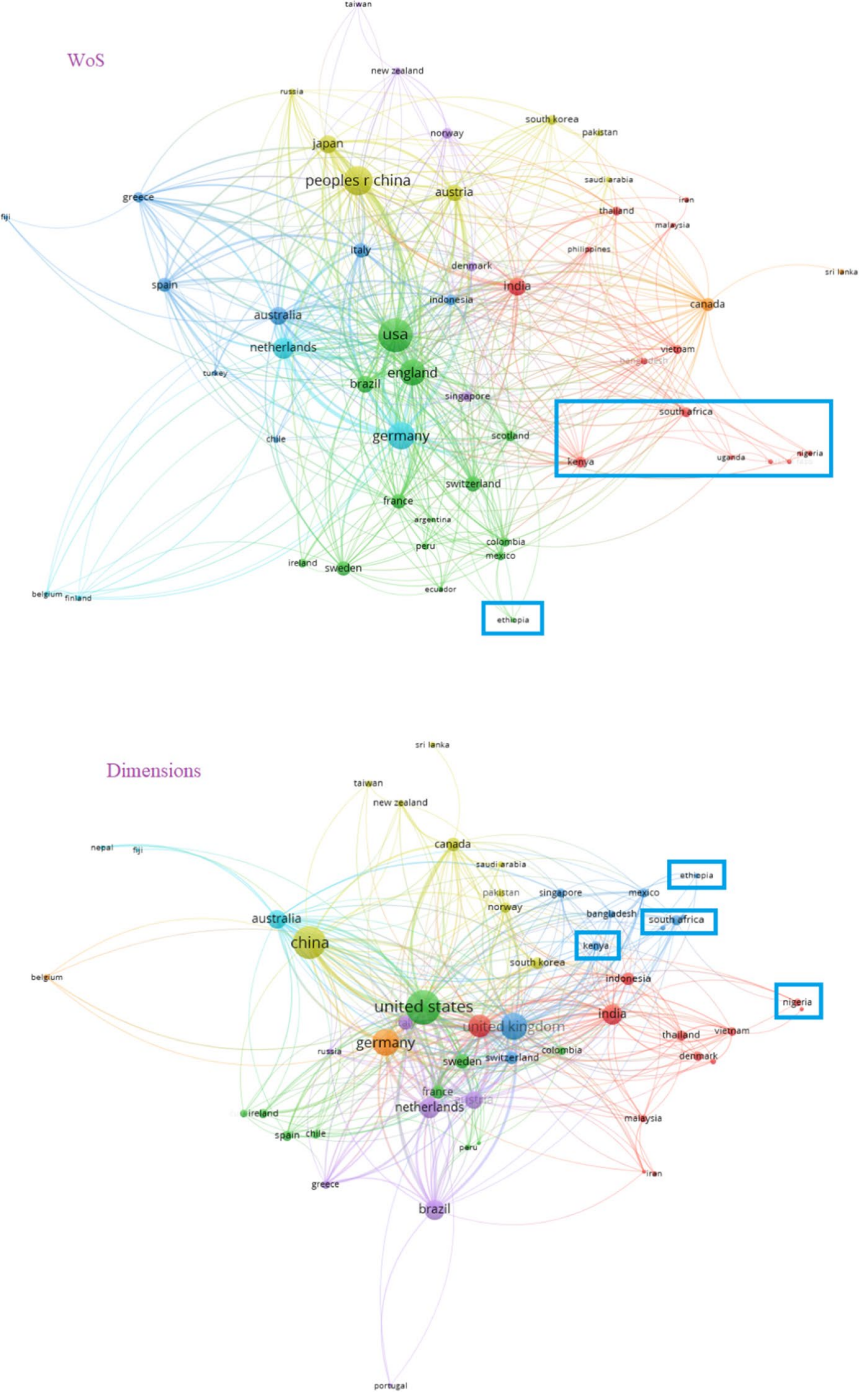
Peer-reviewed literature about NDCs by countries (WoS and Dimensions)

**Fig. 5:**
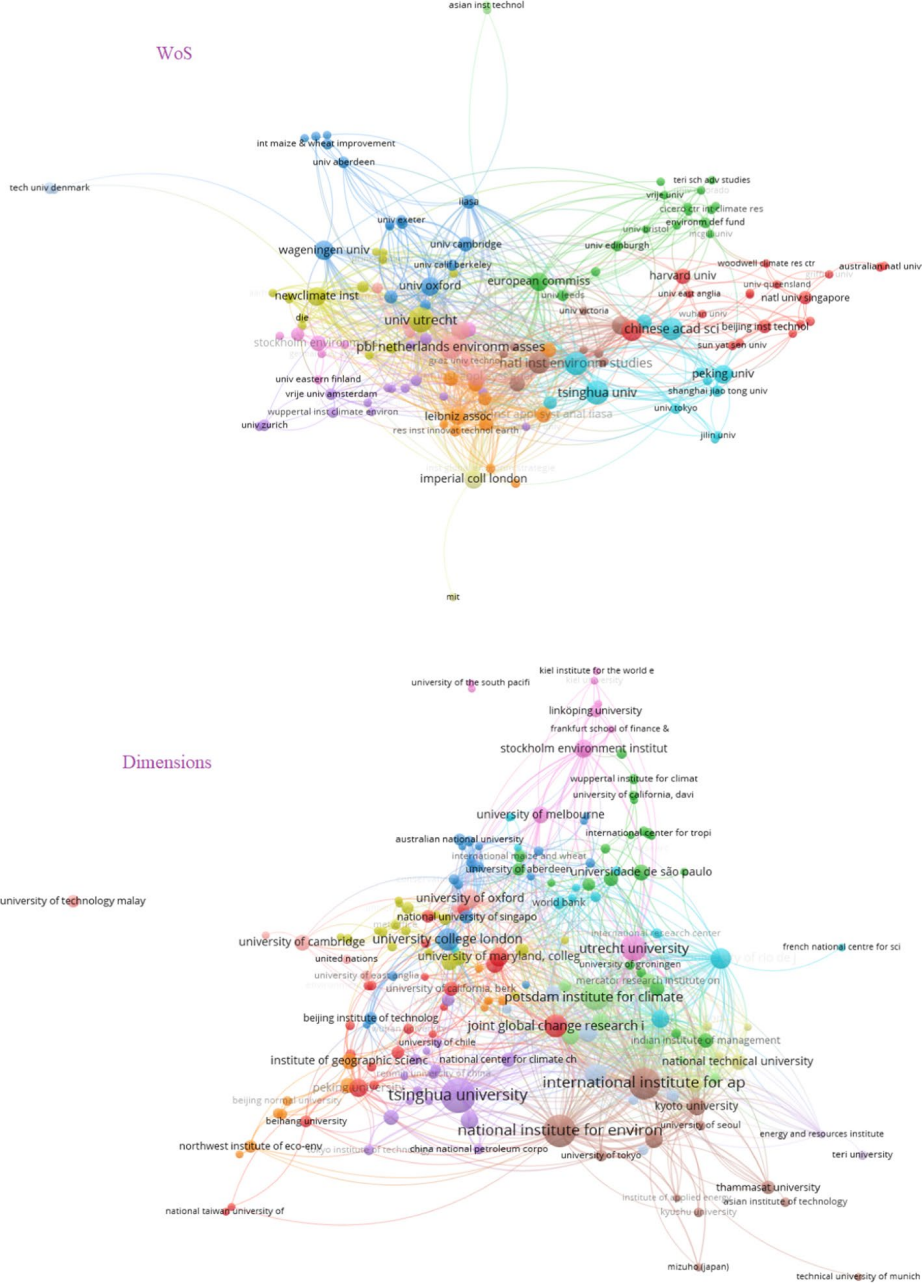
Publications about NDCs by institutions (WoS and Dimensions)

**Fig. 6:**
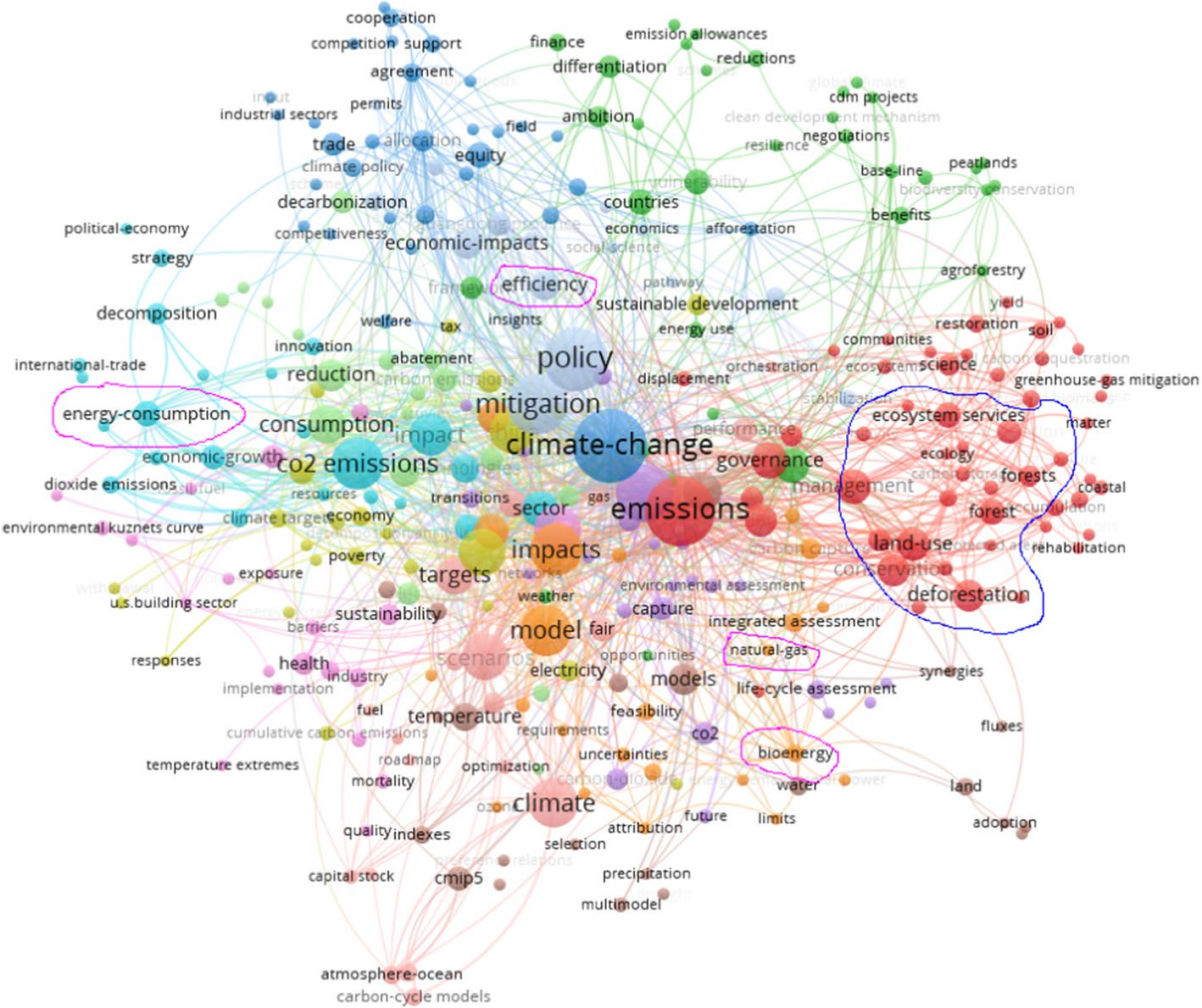
Publications by domain/sectors (WoS)

To determine the trend and spread of NDC research using the “Nationally Determined Contributions”, outputs from WoS and Dimensions were used. In total 406 and 503 articles from WoS and Dimensions were used, respectively. For the trend, the 406 and 503 articles were plotted against the years of publications (2015–2022) as shown in Fig. 3. The same 406 and 503 outputs were used to explore the country and institutions of researchers globally as shown in Figs. 4 and 5, respectively. For the spread by sector, only 406 from WoS was used. Although 503 from Dimensions is far greater than 406 from WoS, it does not allow for the use of keywords of research articles to be used as a unit of analysis. The analysis of the 406 outputs from WoS is presented in Fig. 6.

### Trend of NDC research

The goal in this section is to establish the trend and those involved in NDC studies since its inception in 2015. In order words the goal here is to address the first research question (RQ1) and the second research question (RQ2) of this study. For the trend, the result is presented in Sect. 5.2.1, and for those involved (by country and institution), the results are presented in Sect. 5.2.2.

#### Global trend (2015–2022) [RQ1]

As indicated in Fig. 3, the annual trend in research outputs have been increasing from 2015 to somewhere around mid-2019, with a dip emerges until mid-2020 and marginal increase from thereon and a significant fall since 2021. It is not clear what might have caused the abrupt inversion and fluctuation trend from mid-2019 to 2022. However, it might be partly due to the global COVID-19 pandemic that has affected research outputs from many researchers (Abramo et al., 2022).

#### Global trend by country and by institution [RQ2]

The outputs from analysing WoS (406) and Dimensions (503) articles from the query using “Nationally Determined Contributions” are presented in Figs. 4 and 5.

Based on Fig. 4, most of the outputs have been published in developed countries. The USA, UK and Germany are the top countries with publications on NDCs. To facilitate understanding, these countries have been highlighted in rectangles. Similarly, Fig. 5 reveals most of the institutions that have published on NDCs are from the Western countries. Some of the institutions include University College London, University of Maryland, University of Oxford, University of Cambridge and University of Melbourne. These all institutions in the Global North. On the other hand, the number of publications from developing countries is very few with very low representations from Africa. In fact, only Kenya, Nigeria, Ethiopia and South Africa have publications on NDCs.

### Publication by domain [RQ3]

The aim of this section is to address the third research question (RQ3) of this study. In other words, the section aims to identify common sectors where NDC has been applied. Although 503 from Dimensions is far greater than 406 from WoS, it does not allow for use of keywords of research articles to be used as a unit of analysis. The analysis of the 406 outputs from WoS is presented in Fig. 6.

A further analysis of the 406 articles from WoS reveal the various sectors where the NDC projects have been developed. These include the energy, renewable, energy efficiency, transport, land use, forestry, agroforestry, biodiversity, water and health sectors (Fig. 6). To facilitate understanding, some of the sectors have been included in the blue and pink zones as shown in Fig. 6. Based on Fig. 6, the various sectors can be categorised into forestry/agroforestry and energy/energy efficiency where both have the most frequency and the third consists of research that combines more than one sector.

#### NDC in forestry/agroforestry

The NDCs studies in forestry and agroforestry include Hendri et al. (2021), Mulia et al. (2020), Gurgel et al. (2019), Dencer-Brown et al. (2022), Hong et al. (2022). Hendri et al. (2021). This study aims to simulate carbon management from the forestry sector in West Papua into the long-term low-carbon sustainable development. Mulia et al. (2020) conducted a study that estimated the mitigation potential of agroforestry carbon sequestration in Vietnam using a nationwide agroforestry database and carbon data from the literature. Gurgel et al. (2019) used computable general equilibrium MIT Economic Projection and Policy Analysis model and simulate alternative carbon pricing scenarios (sectoral versus economy-wide carbon markets) to measure the economic impacts of Brazil's climate mitigation strategies contained in its Nationally Determined Contribution (NDC) in the land use and forestry sector. Dencer-Brown et al. (2022) investigated how NDCs can work for both blue carbon and local coastal communities. A study by Hong et al. (2022) used the Korean forest growth model (KO-G-Dynamic model) to analyse various climate change and forest management scenarios and their capacity to address the NDC targets.

#### NDC in energy and energy efficiency

In the field of energy and energy efficiency, a number of peer-reviewed literature exploring how it has been used as part of the NDC initiative exist (Carvalho et al., 2020; Chaichaloempreecha et al, 2019; Herran & Fujimori, 2021; Misila et al., 2020; Vishwanathan & Garg, 2020). In 2019, Chaichaloempreecha et al. (2019) evaluated the Thailand's energy policy on CO_2_ emissions: Implication of National Energy Plans to Achieve NDC Target. This was followed by another by Misila et al. (2020) that analysed the potentials of GHG emission reduction during 2015–2050 from utilisation of renewable energy and increasing energy efficiency using the Long-range Energy Alternative Planning system (LEAP) model for the same country. In Japan, Herran and Fujimori (2021) investigated the energy and macroeconomic impacts of enhancing the ambition of 2040 and 2050 emission reduction targets in Japan by means of a computable general equilibrium (CGE) model. In Brazil, Carvalho et al. (2020) investigated the likelihood of Brazil to achieve its NDC commitments in the energy sector. In India, a study proposed a quantitative assessment using bottom-up optimisation model (AIM/Enduse) to assess how to achieve global GHG mitigation targets of 2 °C and well below 2 °C until 2050 while achieving the national sustainable development goals (Vishwanathan & Garg, 2020).

#### NDC research in multiple sectors

Some of the peer-reviewed studies focused on a combination of sectors. Rathmann et al. (2020) investigated the role the following sectors (AFOLU, industry, agriculture, transport, electric, energy, industrial processes, waste management) can contribute to achieving NDC commitments in Brazil. Köberle et al. (2020) used the Brazil Land Use and Energy System (BLUES) model to explore low-emission scenarios for Brazil for the 2010–2050 period that cost-effectively raise ambition to levels as part of their NDCs. The finding from the study reinforced the fundamental role of the Agriculture, Forestry and Other Land Use (AFOLU) sectors and explore intersectoral linkages to power generation and transportation. Fobissie et al. (2019) examined how Agriculture, Forestry and Other Land Use (AFOLU) activities and their contributions to NDCs are represented in the post-2020 climate change commitments of African countries and assess the necessary conditions for their successful implementation.

While the analysis of results that led to Fig. 6 revealed NDC studies in forestry/agroforestry and energy and energy efficiency, the building sector was noticeably missing. There is very little evidence of peer-reviewed research of NDC applied to buildings. This despite the building sector negatively contributes significantly to the environment and also one of the sectors with the greatest potential to mitigate climate change.

### M&E trends and their common applications in NDC [RQ4]

The aim of this section is to examine the trend of and common M&E systems applied in evaluating NDCs. In other words, the goal here is to address the fourth research question (RQ4) of this study. To facilitate understanding, the constituent of an M&E system will be summarised here. According to Vallejo (2017), the M&E system should include things like who is responsible for M&E tasks in the organisation, the intervals where data should be collected, how the data is collected, who collects the data, the type of database that is used for storing the data, the standard forms and data collection tools to be used, how the data is analysed, the evaluation questions, the frequency with which an evaluation takes place, the budget allocated for evaluation, etc. These requirements have often been represented in tabular forms to facilitate understanding and readability. Excellent examples of M&E templates have been developed by Bullen (2018).

Using the search terms from row 5th “Monitoring and evaluation” and “Nationally Determined Contributions” to 15th “M&V” and “Nationally Determined Contributions” for Dimensions and WoS, the outputs were insignificant which limited an investigation of the trend of M&E applied in NDCs. Consequently, the search was expanded to include ScienceDirect. Although the results from Science Direct were quite sizeable, on detail analysis, only 5 articles discussed M&E applications on NDCs (Visman et al., 2022; Meehan et al., 2019; Gonçalves et al., 2019; Leon & Izumi, 2022; Usapein & Chavalparit, 2017).

Visman et al. (2022) propose a framework that take into account various approaches for monitoring and evaluation of both the process and outcomes of investments in climate services co-production, so that scientific excellence can be monitored alongside development impact. Meehan et al. (2019) evaluated the design and implementation of the Indonesian’s first National Action Plan for Greenhouse Gas Emissions Reductions which reflects its emission reduction commitments to 2020. Gonçalves et al. (2019) developed and modelled scenarios of energy use and GHG emissions from the transport sector until 2030 for meeting the Brazilian Nationally Determined Contribution. To increase the adoption of alternate wetting and drying to fulfil NDCs, a study was conducted by Leon and Izumi (2022) to evaluate the impacts of alternate wetting and drying on profit and life cycle greenhouse gas emissions throughout the year. In order to achieve this survey, data were collected by a structured interview from two groups of farmers in An Giang Province in Vietnam. Usapein and Chavalparit (2017) developed an MRV guidelines for the Thailand Voluntary Emission Trading System (V-ETS) so that factories participating in the system can effectively, consistently, reliably and compatibly report their GHG emissions. It emerged that none of the aforementioned articles has elaborately examined any M&E systems such as log frame, performance indicators, institutional arrangements, etc. This lack of details limits understanding of how M&E can be applied in NDCs.

Upon noticing the weaknesses with M&E for NDCs peer-reviewed literature, the focus was turned to grey literature (highlighted in Fig. 2) where an analysis of the M&E systems on climate change related projects for some selected countries have been conducted. The included grey literature about projects in South Africa (RSA, 2018), Ghana (Benefoh & Amoah, 2017), Cote d’Ivoire (Assamoi, 2020), Morocco (CBIT, 2018), Mongolia (Akker & Jargal, 2020) and Mongolia (FAO & UN, 2019). For the case of South Africa, the National Climate Change Response M&E system is the main system to track South Africa’s transition to a lower-carbon and climate-resilient economy and society (RSA, 2018). The Department of Environmental Affairs is the institution leading this project. In Ghana, an MRV system has been put in place for the a) continuous data measurements & collection on GHG emissions, mitigation actions, climate support, track progress of NDC targets, preparation & compilation, b) domestic disclosure and international reporting and c) technical and “policy” review (verifications) (Benefoh & Amoah, 2017). The agencies involved in this are the Ghana Statistical Service, National Developing Planning Commission, Ministry of Local Government and Rural Development, Ministry of Food and Agriculture (SRID) and the Environmental Protection Agency. In Cote d’Ivoire an MRV system has been developed. The core of this system is an adaptation metric developed from Notre Dame Global adaptation index for measuring the vulnerability of Cote d’Ivoire with respect to climate change (Assamoi, 2020). The project is part the UNDP-UN Environment National Adaptation Plan Global Support Programme (NAP-GSP). In Morocco, its government started developing an integrated transparency framework for NDC planning and monitoring in 2018 (Morocco, CBIT, 2018). The organisations involved in this effort are the UNDP, State Secretariat of Sustainable Development of Morocco. In Mongolia, an M&E system was used to evaluate Nationally Appropriate Mitigation Actions in the Construction Sector in Mongolia (Akker & Jargal, 2020). The institutions involved are the Global Environment Facility (GEF) and the Ministry of Construction and Urban Development (MCUD). Another initiative in Mongolia is an improved monitoring and reporting system for the correct implementation and tracking of NDCs (FAO & UN, 2019). The agencies involved are the Ministry of Environment and Tourism and the Climate Change Project Implementation Unit. The grey literature revealed the context of each country influenced their choices of M&E systems NDC projects. This led to M&E not to use on all NDC projects. Some authors have argued the implementing M&E can be difficult. In Australia, Scott and Molony (2022) found that monitoring and evaluation of climate change adaptation is challenging for local governments.

## Limitations and challenges

In this study, Theofanidis and Fountouki (2018) research has been used as a lens through which to identify the limitations. The authors define limitations as any potential weaknesses usually out of the researcher’s control and are closely associated with the chosen research design, statistical model constraints, funding constraints or other factors. To this end, three main limitations were identified.

Firstly, some articles did not have NDC in their titles, abstracts, content or body, yet they clearly can inform sustainable development projects and more specifically NDC goals (Barry & Hoyne, 2021; Defe & Matsa, 2021). Barry and Hoyne (2021) and Defe and Matsa lack traces of NDC in them, yet they have addressed issues relevant to NDC. There is a possibility that this study missed the articles that fall into the aforementioned category.

Secondly, some articles that were/are review types could have been inadvertently considered in the analysis that led to the development of Table 1 and Figs. 3, 4, 5 and 6. For example, Praene et al. (2021) is not classed as a reviewed article in ScienceDirect although the content and methodology of research shows it is a review article. This is because although most review articles contain the word “review” in their titles and/or stated in their abstracts, some do not. On the other hand, some articles are classed as review when in fact they are not review articles. This meant there was a possibility of missing some articles not included in Table 1 that could have been pertinent to this research. An example is Sani et al. (2021), which is classed as a review article in Science Direct, although the content shows otherwise. This is tedious to the researchers who had to spend time browsing and reading these kinds of articles to make sure they meet the criteria of being peer-reviewed articles as one of the inclusion criteria of this study. Despite this effort one cannot conclude review articles were totally excluded from the considered articles used in WoS, Dimensions and Science Direct.

Lastly, some short forms adopted in the search brought out results that were entirely not relevant to this study. For example, some searches yielded outputs such as Li et al. (2022) because it contained the chemical name nitrogen-doped carbonised wood (NDC). This NDC as used in this article does not stand for Nationally Determined Contributions. Despite the authors efforts to manually filter these kind articles, it cannot be ascertained that the final output (Table 1) used for the analysis was entirely free of similar articles.

## Recommendations and directions of future research

### Recommendation 1: Future research should be more inclusive

Figures 2 and 3 indicate that most peer-reviewed articles were published by institutions in the global North. Given the impact of NDCs in the global South being far greater than that of the global North voices from the global South are important for efficient mitigation and adaptation strategies. A quote from Dr Marton Demeter—a researcher at the National University of Public Service in Budapest with a specialism in “publication networks” (Tandon, 2021) reads:

“Diverse communities, including academic communities, can provide more creative work. Research shows that the contributions of diverse research groups are generally better than those of their less diverse counterparts.”

As a recommendation, a detailed study that is more inclusive, considering many relevant voices and factors such as number and origin of lead authors, gender disparity, etc., should be undertaken to inform policies on how to fund climate change research. This may contribute to stopping the practice of “helicopter science” and encourage more involvement of developing countries’ scientists in climate change research.

### Recommendation 2: A more holistic approach covering more sectors

The peer-reviewed literature reveals the disparity in the levels of NDC in the different sectors. Perhaps this is due to the fact that, as argued by Atteridge et al. (2020) most NDC actions relate generally to themes in the respective national development plan, but the range of sectors they include as climate priorities is narrow and does not reflect the spread of national development agendas. This is also noticeable in the technology needs assessment reports whereby, countries using multi-criteria decision analysis to identify priority sectors or NDC measures. While this practice is efficient an effective, a broader perspective of considering all measures that can provide better insights about impacts especially given the many sectorial dependencies can often be missed. Although some intersectoral studies exist (Fobissie et al., 2019; Köberle et al., 2020; Rathmann et al., 2020) as discussed in Sect. 5.3.3, their focus is on AFOLU. Perhaps, this is partly because AFOLU is often considered as a block in most international reports such as the Intergovernmental Panel on Climate Change (IPCC). However, some of the AFOLU sectors are interlinked with other non-AFOLU sectors. For example, a building (building sector) located for away from a farm (agriculture) may lead to high transport (transport sector) emissions when transporting crops to the farmer’s building or markets in the cities. As a recommendation, future studies should expand to include many sectors especially those that have not featured regularly in published reports. This may potentially contribute to informing sectorial or NDC measures prioritisation studies. One such sector that have featured less in TNA reports (TNA, 2022) and peer-reviewed research (deduced from Fig. 6) is the building sector. Despite being one of the sectors with the highest impacts on the environment and also being one of the sectors with the greatest potential to mitigate climate change impacts, there is paucity of peer-reviewed and grey literature on NDC measures from the building sector. Also, despite a study by Powell et al. (2018) underscoring the fact that the research about the contribution of the waste sector towards NDCs, there is still no evidence of increase in research in the sector.

### Recommendation 3: More integration of M&E applied in NDCs

M&E is a well-established discipline in the aids’ development fields. Research about M&E applied to aids development projects and programmes are quite common (Abanda et al., 2021). Similarly, there is an abundance of grey literature about M&E of NDC measures. However, the situation is different with peer-reviewed literature lacking about M&E applied on NDC measurers. This is evident with very low search results obtained from row 5 to row 15 of Table 1. Moreover, the grey literature is mostly in the form of reports financed by donor agencies. The fact that the reports receive funding from donor agencies makes the M&E report outcomes a bone of contention between evaluators, donor agencies and/or policy-makers which instil mistrust in the hearts of community members. Edmunds and Marchant (2008) have argued that one cannot expect unbiased analysis to be conducted in M&E studies unless the evaluators are themselves independent of the programme they are evaluating. This is particularly true when policy-makers have a vested interest in framing these outcomes in a positive light—especially when they previously expressed a commitment to the reform (Vaganay, 2016). This study recommends future studies should focus on empirical investigations of M&E applications on NDCs. Not only will this instil trust in organisations and researchers with interests in M&E for NDCs, the empirical peer-reviewed study may provide opportunities to corroborate findings from published grey reports by donor agencies.

## Conclusions

The motivation for this study is the fact that, despite developing countries suffering from the burden of climate change impacts, it was not clear to what extent their voices are considered in climate change research. The study focused on NDCs and their evaluation using M&E systems. To this end, a review of the literature was conducted to establish the problem domain and identify the knowledge gaps. Building on this, a systematic review of the literature was conducted using WoS, Dimensions and ScienceDirect databases. The first finding revealed a surge in published research from 2015 since the inception of NDC, although the trend became very unpredictable from mid-2019 until date. The second finding of the research revealed a global North–South disparity in the number of research institutes and researchers involved in NDC research. Thirdly, there is a global paucity of peer-reviewed research about M&E of NDC measures. Although significant progress has been made in terms of research in the global North, Scott and Molony (2022) found that monitoring and evaluation of climate change adaptation is challenging for local governments in Australia. While countries in the global North are conducting research about NDCs and M&E for NDCs, it should be as inclusive and diverse as possible involving researchers in the global South not only as research assistants for data collection and local guides but playing major research roles. Taking into account these research findings and aforementioned recommendations, this study has implications on policy, research and practice. From a policy perspective, it can serve as the basis for donor agencies and governments to commence elaborating the steps in overcoming the South–North divide in climate change research especially with regards to NDCs. From a research perspective, directions of future research have been indicated which can underpin the basis for future research by other researchers. From a political perspective, while pressure on highest CO_2_ emitters to roll out more NDC projects is necessary, research into furthering knowledge about NDC should continue and should be as inclusive as possible capturing every voice especially those from developing countries. Lastly, from a practice perspective, the uptake of research in NDCs and M&E of NDCs can lead to the enhancement of capacities of professionals in the field of M&E with a focus on NDCs. This is particularly important, given many countries will be proposing their 2nd and possibly 3rd NDCs in the future. In fact, there are already calls for countries to submit more ambitious NDCs during the next CoP 27 to be held in Egypt from 7 to 18 November 2022 (United Nations Climate Change, 2022). M&E will be crucial in the successful implementation of NDCs by various governments.

## Data Availability

The data supporting the findings of this study are available within the article.
